# Diagnosis, Treatment, and Follow-Up of Tracheo/Bronchomalacia in Children: The Italian Multicenter Experience

**DOI:** 10.3390/children12111511

**Published:** 2025-11-07

**Authors:** Angelo Florio, Michele Ghezzi, Francesca Rizzo, Paolo Del Greco, Katia Perri, Fabio Antonelli, Annalisa Gallizia, Francesco Santoro, Elena Ribera, Francesco Macchini, Michele Torre, Francesco Donati, Federica Lena, Vittorio Guerriero, Paola Borgia, Valerio Gentilino, Roberto D’Agostino, Federica Porcaro, Alessio Conte, Duino Meucci, Roberto Baggi, Michele Gaffuri, Pietro Salvati, Oliviero Sacco

**Affiliations:** 1 UOC Pneumologia Pediatrica ed Endoscopia Respiratoria IRCCS G. Gaslini, 16147 Genova, Italykatiaperri@gaslini.org (K.P.); paolaborgia@gaslini.org (P.B.);; 2 Clinica Pediatrica, Ospedale dei Bambini V. Buzzi, 20154 Milano, Italy; 3 UOC Radiologia IRCCS G. Gaslini, 16147 Genova, Italy; 4 SOSA di Broncopneumologia, AOU Meyer, IRCCS, 50139 Firenze, Italy; paolo.delgreco@meyer.it; 5 UOC Pneumologia e UTSIRA.O.P. Santobono-Pausilipon, 80129 Napoli, Italy; f.antonelli@santobonopausilipon.it; 6 UOC Cardiochirurgia IRCCS G. Gaslini, 16147 Genova, Italy; 7 S.C. Chirurgia Pediatrica GOM Niguarda, 20162 Milano, Italy; 8 UOSD Chirurgia Toracica e vie Aeree, IRCCS G. Gaslini, 16147 Genova, Italy; micheletorre@gaslini.org (M.T.);; 9 S.C. Chirurgia Pediatrica, Ospedale Filippo Del Ponte, 21100 Varese, Italy; 10 U.O.C. Otorinolaringoiatria, IRCCS G. Gaslini, 16147 Genova, Italy; 11 UOC Pneumologia e Fibrosi Cistica, Ospedale Pediatrico Bambino Gesù, IRCCS, 00165 Roma, Italy; federica.porcaro@opbg.net; 12 DINOGMI Università degli Studi di Genova, 16132 Genova, Italy; s4030412@studenti.unige.it; 13 UOS Chirurgia Delle Vie Aeree—UOC Otorinolaringoiatria, Ospedale Pediatrico Bambino Gesù, 00165 Roma, Italy; duino.meucci@opbg.net; 14 SOSA Tracheal Team Azienda Ospedaliero-Universitaria Meyer, 50139 Firenze, Italy; 15 S.S. Otorinolaringoiatria Pediatrica, Fondazione IRCCS Ca’ Granda Ospedale Maggiore Policlinico di Milano, 20122 Milano, Italy

**Keywords:** tracheomalacia, spirometry, videobronchoscopy, anterior aortopexis, posterior tracheopexis, esophageal atresia (EA), TEF endoscopic treatment, tracheal splint, tracheal stent, NIV in tracheomalacia patients

## Abstract

Background: In pediatric age, the central airways are more flexible and mobile, with tracheal and bronchial walls easily tending to collapse, allowing partial or complete occlusion of the lumen: a situation described as tracheobronchomalacia (TBM). This is a condition that causes an increase in intrathoracic pressure that may accentuate airway collapse, and a biphasic or barking cough appears. Objectives: Although TBM is relatively frequent in pediatric age, the diagnostic criteria and subsequent treatment do not follow well-standardized criteria and often vary from pediatric center to center. Therefore, there is a need to standardize diagnostic procedures and the resulting medical or surgical treatments. Methods: We therefore organized a day of meetings to talk about TBM, inviting all Italian pediatricians and pediatric surgeons who diagnose and treat patients with this pathology on a daily basis. Results: This work, collecting all the meeting interventions, is a compendium that deals with all aspects of TBM, emphasizing the most correct criteria to diagnose and therefore best treat each pediatric patient with this clinical condition. We give particular emphasis to the need to perform static and dynamic videobronchoscopy (S/DVBS) to verify the patency of the tracheal lumen, so as to evaluate the severity of TBM. Conclusions: this work deals with TBM in all its diagnostic and treatment aspects and can be a valid help for all pediatricians who treat these patients.

## 1. Introduction

Tracheobronchomalacia (TBM) is a relatively frequent clinical condition in pediatric patients, and it is important to diagnose, especially in pediatric age, because it hinders the normal clearance of bronchial secretions, so it is easily associated with clinical complications that can then evolve into a chain of events. The first complication is represented by recurrent/persistent respiratory infections, which predispose patients to the onset of protracted bacterial bronchitis, characterized by the persistence of a bacterial load in the deep lung even in conditions of relative well-being. The persistence of infection and inflammation at the bronchial wall level over time then causes wall damage with the formation of bronchiectasis, particularly in the lower lung lobes.

Despite this, the diagnostic criteria and treatment of TBM are still highly debated and not standardized, so they can vary greatly from different pediatric centers.

Furthermore, clear international guidelines that can be applied to pediatric patients with this pathology are missing, especially regarding the selection criteria of patients deserving of surgical therapy: anterior aortopexy or posterior tracheopexy. The 2019 ERS statement on tracheomalacia and bronchomalacia in children also deals with these surgical procedures in a very brief manner and does not outline clear clinical and diagnostic criteria for selecting patients to be surgically treated.

We organized a day of discussion among all Italian pediatric centers that treat this pathology; the different speakers covered their fields of experience, in which they are well recognized as authorities for their experience and clinical case studies: pulmonologists, endoscopists, pediatric surgeons, and otolaryngologists. This article precisely derives from all the interventions and consequent discussions that were held on 8 June 2024 at the IRCCS G. Gaslini Children Hospital (Genova, Italy), with the intention of covering all aspects that may be of interest to doctors dealing with this pathology.

This work is divided into various sections, starting from the clinical history, which must lead to suspect TM. The diagnostic section is as follows: radiographic, spirometric, and endoscopic tests, giving particular importance to the need for bronchoscopy to be dynamic (performed with a phase in which the patient also performs abdominal straining), to verify how the tracheal lumen changes with the increase in intrathoracic pressure. Once the diagnosis of TBM has been made and the case discussed within a multidisciplinary team, if surgery is indicated, the most suitable treatment for every single patient is identified. A section is dedicated to the most severe cases of malacia: TM associated with esophageal atresia. The follow-up of patients with TM is next, which underlines the need to have a multidisciplinary team available, considering the various pathologies that are often associated with TM patients. The final section is about the respiratory assistance of the TM patients, as a bridging therapy while waiting for surgery, or when this is not feasible.

A general discussion was held at the end of each section, highlighting how there is still no unity of opinion between the various Italian pediatric centers. The indications for surgical treatment were the most controversial topic: some centers had a more conservative attitude, while others placed indications for TM surgery earlier in the clinical history, with the aim of anticipating and preventing the appearance of infectious complications as protracted bacterial bronchitis.

We recognize that the final result of all this is not easily labelable; the best definition that can be attributed to our work is the Italian multicenter experience, as stated in the title. We hope this work could be the basis for a future national guideline on the diagnosis and treatment of TM, stimulating broader knowledge about the best diagnosis and treatments of this pathology.

## 2. The Clinical History and the Typical Two-Tone/Biphasic Cough of Patients with Tracheomalacia (TM)

In pediatric age, the central airways are more flexible and mobile, tending to anteroposterior diameter (APD) collapse, with partial or complete occlusion of the tracheal lumen [1]. The trachea is surrounded by mediastinal vascular structures: the aortic arch and epiaortic vessels, and its lumen can be easily narrowed by extrinsic vascular compression. All congenital anomalies of the aortic arch, present in 1–2% of the general population, such as the right aortic arch, aberrant Innominate Artery (IA), complete vascular rings with double aortic arch and left pulmonary artery sling, are all conditions causing extrinsic tracheal compression [2]. Even in patients with primary TM (i.e., without extrinsic vascular compression), all conditions cause increased intrathoracic pressure; cough, crying, Valsalva maneuver, forced expiration, and physical exertion can accentuate airway collapse and the appearance of biphasic or barking cough (brassy cough/barking seal cough) in 70–80% of cases [3].

The tone of the cough is biphasic because the first tone (physiological) is generated by the larynx, and the second (pathological) is caused by the tracheal wall vibrations when it collapses, up to complete occlusion of the lumen, at the level of compression/malacia. The biphasic cough can be easily recognized by the human ear because the harmonics of the sound coming from the larynx and those generated by the tracheal wall have different frequencies (Hz) [4,5].

In the diagnostic pathway, if the patient at the time of the visit is not already coughing with a biphasic cough, it is important to ask parents the following:

- “Does it seem to you that your child has a different tone of cough than his/her peers?”
- “Would you be able to recognize your child’s cough in different contexts?”

In many patients, a biphasic cough occurs only in conjunction with a respiratory infection when they are coughing aggressively, which can contribute to a delayed diagnosis. This clinical condition should also be considered in patients with recurrent wheezing or those mistakenly labeled as having allergic asthma, not responding to usual inhalation therapy [6]. Reduced exercise tolerance and exertion-induced cough can also be symptoms present in patients with TM: the increased intrathoracic pressure worsens airway collapse, so that even these patients are frequently misdiagnosed as asthmatic [7]. Severe TM can be clinically evident from birth, characterized by cyanosis and/or stridor, even in quiet breathing; cardiac arrest or sudden infant death may rarely occur. These patients require intubation and are then difficult to extubate, unless surgically treated by removing the vessel compressing the trachea, if malacia is secondary to extrinsic compression.

Airway collapse during expiratory efforts and consequent ineffective coughing can cause altered secretion clearance with increased risk of recurrent respiratory infections and persistent productive cough: clinical picture suggestive of protracted bacterial bronchitis (PBB) [8,9]. In Kompare’s study, in a population of 70 patients with PBB, tracheobronchial malacia (TBM) was present in 74% of them, so it is important to think about TBM, especially in patients with PBB [8,9].

In the presence of a biphasic cough, particularly if associated with recurrent/persistent respiratory infections, second-level diagnostic investigations have to be performed: chest Computed Tomography (CT) with Contrast Medium (CM) plus S/DVBS [10,11,12]. After these investigations, every patient is then discussed within our institute’s Tracheal Team (TT) for case-by-case therapeutic decisions.

**Discussion**. All the participants underlined the importance of recognizing the two-tone cough as the most important clinical sign suggesting TM. It was also emphasized that the biphasic cough generally appears after two years of age, when the patient is strong enough to generate a significant increase in intrathoracic pressure, causing the collapse of the tracheal lumen if TM is present. The clinical history must be particularly accurate regarding the recurrence of lower respiratory tract infections every time TM is suspected.

## 3. Diagnostic Tests

### 3.1. Imaging Techniques

The videobronchoscopy (VBS) is the “gold standard” for the diagnosis of TM and TBM, but the usefulness of various imaging techniques has been demonstrated, so as to integrate the following endoscopic data:

- Chest radiography (CXR): It is not useful for the diagnosis of TBM, although it can visualize the tracheal lumen, which may be focally slightly lateralized in cases of extrinsic compression from the aortic arch.
- Tracheobronchography–fluoroscopy (TBG-TBF): This is often performed concurrently with endoscopy in the operating room under general anesthesia. Iso-osmolar CM is injected through the working channel of the bronchoscope, obtaining an immediate and panoramic evaluation of the airways. Data are obtained regarding airway dimensions (even downstream from stenosis), morphology (normal or abnormal bronchial bifurcation, vs. isomerisms), as well as changes in tracheal lumen during different phases of respiration. TBG is also very useful as a guide for subsequent interventional procedures, favoring precise luminal localization of devices (balloon tracheoplasty, cutting balloon, bioabsorbable stent) [13,14,15].
- Esophagography–fluoroscopy (EG-EF): This is performed for searching tracheoesophageal fistulas (TEF), often present in esophageal atresia (EA) and frequently associated with TM. Diagnostic accuracy for some authors is >80%, while for others this is not an effective technique to reveal the fistula [16]. CT can demonstrate the presence of the TEF, but definitive diagnosis on patency or closure of the fistula is certainly entrusted to endoscopy with direct injection of CM into the fistula through the working channel of the endoscope.
- Computed Tomography (CT): This is a continuously evolving imaging technique, rapid and non-invasive, providing an excellent overall view, independent of body size, with high spatial/temporal resolution. It allows multiplanar and volumetric reconstructions (MPR, MinPR, MipPR, and Volumetric 3D). It can be performed on children of all ages; anesthesia/sedation may be necessary under 5 years of age. Flash Monophasic Technique performed with a single scan, after intravenous injection of CM, provides information on airway morphology (but not dynamics), visualizing airways even distal to the site of obstructions and on mediastinal vessels exerting compression on the trachea or bronchi, highlighting any mediastinal pathology. CT shows cardiovascular anomalies compressing the airway, such as right aortic arch, complete/incomplete double aortic arch, pulmonary sling, and aberrant IA, all causing more or less severe TBM [16]. TM is very frequently associated with EA; CT can demonstrate malacia and extrinsic tracheal compression with a significant reduction in the tracheal ADP at the point of intersection with IA. CT can also demonstrate irreversible lung damage, such as bronchiectasis formation, caused by chronic recurrent lung infections resulting from reduced mucociliary clearance in TBM. Skeletal anomalies (e.g., pectus excavatum and scoliosis) that can cause airway compression and consequent TBM are also demonstrated. CT also evaluates tracheal compressions caused by space-occupying mediastinal lesions. Virtual bronchoscopy obtained with 3D airway reconstruction on CT images has not been very sensitive (<75%) in detecting TBM [17,18].
- Dynamic CT (DCT) allows visualization of the entire airway in a single gantry rotation using a dynamic volumetric scanning technique. Images can be acquired over one or two respiratory cycles with a total scan time of less than 2 s, while the child is breathing at tidal volume during this rapid acquisition. Anesthesia is not necessary, making the exam comfortable and drastically reducing patient discomfort. Dynamic imaging throughout the respiratory cycle allows accurate determination of end-inspiration/end-expiration phases in three dimensions (3D), with accurate determination of luminal collapse degree. The disadvantage of this technique is increased radiation dose to the patient, so it is performed only in selected cases [19,20].
- Magnetic Resonance Imaging (MRI) is the exam of choice for studying cervical or thoracic masses compressing or displacing the trachea in pediatric age (cervical lymphangioma, venous vascular malformation, neuroblastoma, esophageal duplication, lymphoma, and teratoma). The main advantage of MRI is the lack of exposure to ionizing radiation, while the disadvantages are the long acquisition times and the lower spatial resolution than CT. Dynamic MRI for tracheomalacia, using three high-field Tesla (3.0 Tesla or 3T MRI) scanners and dedicated coils, is still limited to the research field rather than clinical application. The time-resolved sequence can demonstrate the dynamic aspect of the airway during forced expiration [1,21].

In conclusion, we believe that the “gold standard” for diagnosing TBM is S/DVBS, but this diagnostic procedure should be supported by other imaging techniques. Among these, CT with CM is the technique that provides the best high-resolution imaging of airways, vessels, and lung parenchyma, as well as the vascular or skeletal anomalies associated with TBM. Third-generation equipment has achieved a reduction in radiation dose and amount of CM used, decreased need for anesthesia/sedation, and generally shortened exam execution times. DCT provides information on airway caliber changes during in and ex-spiration cycle, but due to the higher radiation dose required compared to Flash Monophasic CT, it is used only in selected cases.

### 3.2. Spirometry

Spirometry/Pulmonary Function Test (PFT) is the most standardized test for evaluating respiratory function in both adults and children when the patient is cooperative. Airway obstructions can occur at both the extrathoracic level (pharynx, larynx, and the initial extrathoracic portion of the trachea) and at the intrathoracic level (intrathoracic trachea and main bronchi). The best test for the diagnosis of TM has not yet been well defined; many studies show that patients with TM present an apparent obstructive pattern on PFT, associated with a Flow/Volume Curve (F/VC) morphology tending to plateau in the expiratory phase [1,22].

In the case of a tracheal obstruction, the most diagnostic functional parameters pertain to the expiratory phase: primarily a reduction in Peak Expiratory Flow (PEF) and, in cases of severe obstructions, in Forced Expiratory Volume (FEV) exhaled in the first second of forced expiration (FEV1) [23]. However, it should be noted that clinical symptoms are often not directly proportional to the severity of spirometry data, and mild TM can often be present even when PFT is almost normal.

**Figure 1:**
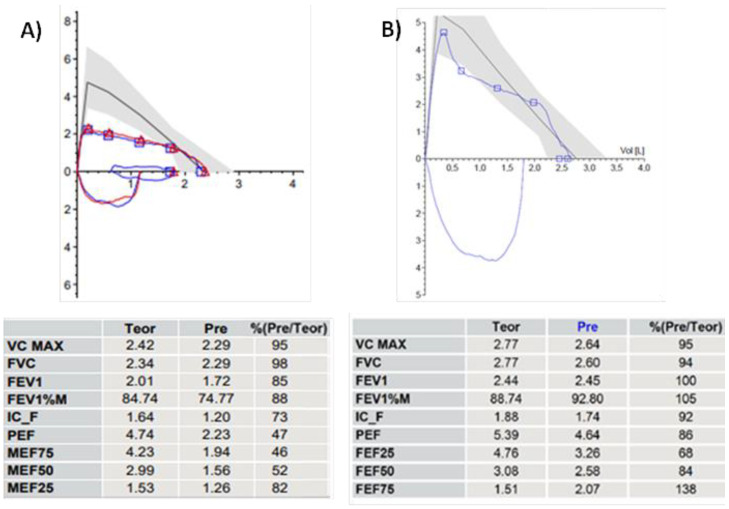
A 10-year-old girl with a hitherto undiagnosed balanced double aortic arch. The patient had always had dyspnea with even moderate physical activity and dysphagia for elastic foods such as meat boluses. (**A**) PFT before and (**B**) 14 months after vascular ring opening surgery, performed a few days after the first PFT. The change in the morphology of the F/VC is impressive, going from a box shape to an almost normal morphology with a PEF value that increases from 47% to 86% of the theoretical values after surgery. **Abbreviations.** PFT: Pulmonary Function Test; F/VC: Flow/Volume Curve. PEP: Peak Expiratory Flow.

**Figure 2:**
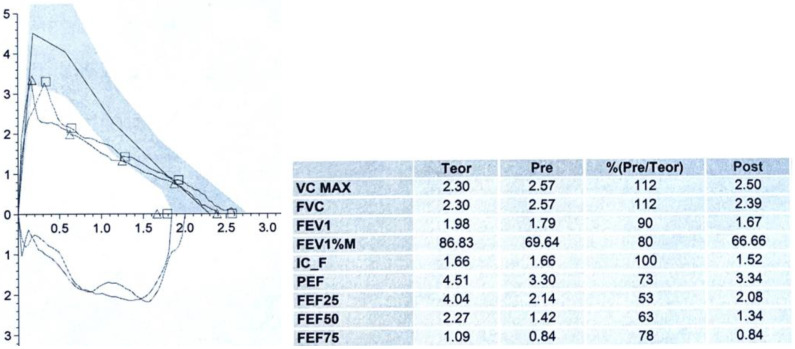
A 9-year-old boy with a two-toned barking cough present, since the age of 2 years, during intercurrent infectious events and physical activity, like running in football matches. The CT scan with CM and VBS showed anterior tracheal compression from the IA with a decrease in the tracheal lumen of approximately 50%. PFT demonstrates normal lung volumes, and the expiratory curve has a reduced PEF: A total of 73% of the theoretical value, and, immediately after the peak, a clear incision is present with a sharp decrease in FEF25, FEF50, and FEF75: 53, 63, and 78% of the theoretical value, respectively. No response to bronchodilator. The indication to perform OAA surgery was not accepted by the parents. The patient was then treated with topical corticosteroids during the infectious events, with a poor clinical response to the recurrent barking cough. **Abbreviations.** CT: Computed Tomography; CM: Contrast Medium; VBS: Videobronchoscopy; IA: Innominate Artery; PFT: Pulmonary Function Test; PEF: Peak Expiratory Flow. FEF: Forced Expiratory Flow; OAA: Open Anterior Aortopexy.

In case of intrathoracic TM, the endotracheal airway pressure during forced inspiration is greater than pleural pressure, so that the inspiratory portion of the flow–volume curve more easily appears to be normal. Conversely, during forced expiration, when pleural pressure is greater than endotracheal pressure, this differential pressure will reduce airway diameter, especially at the level of malacia. This results in an increased obstruction, corresponding to a flattening/decapitation of the expiratory portion of the F/VC, with a marked reduction in Peak Expiratory Flow (PEF): Figure 1A. Last, but not least, PFR data and F/VC morphology in patients with TM show no change after bronchodilation [24]: Figure 2.

### 3.3. Static and Dynamic VBS (S/DVBS): Endoscopic Pictures in Clinical Cases

TM can occur due to the following:

1. Primary malacia: This is due to intrinsic alteration of the cartilage of the respiratory tract walls, and it is a less common condition compared to the following:
2. Secondary malacia: This is the normally shaped cartilage that is deformed by extrinsic compression, usually of vascular origin, on the tracheal wall.
3. Hypermobility of the posterior wall: This is when the pars membranacea protrudes exaggeratedly/pathologically into the tracheal lumen during expiration and cough [25,26].

All these conditions, which can also coexist, result in a variable reduction in the tracheal lumen. The ERS statement [1] defines the severity of TM as a percentage reduction, calculated as a ratio of the diameters of the tracheal lumen in the non-malacic area, evaluated subjectively by the bronchoscopist during tracheoscopy.

- Mild TM: reduction between 50–75%;
- Moderate TM: reduction between 75–90%;
- Severe TM: reduction is >90%.

This stratification of the degree of TM is flawed, first due to the fact that the endoscopic evaluation can be very subjective, and, secondly, due to the fact that the lumen reduction is judged during quiet breathing, without considering that the trachea is not a rigid tube but has elastic and yielding walls, especially in children, so its patency can greatly change during the respiratory cycle and especially when the patient coughs.

In our opinion, VBS performed in pediatric patients in the operating room under sedation should be as follows:

- Static VBS (SVBS) is the phase in which the tracheal lumen is evaluated during spontaneous, quiet breathing, but there should also be a phase of:
- Dynamic VBS (DVBS), in which the patient (while sedation, as it is at the end of the examination, becomes increasingly superficial), is stimulated to cough by contact of the endoscope with the carina and/or main bronchial walls. The patient, performing abdominal straining to attempt to cough, increases the intrathoracic pressure similarly to what happens when the patient is awake. The increasing pressure, if there is malacia/extrinsic compression or hypermobility of the pars membranacea, causes a pathological (i.e., greater than 50%) decrease in tracheal lumen, as can happen in everyday life [27,28].

To better understand why VBS should also have a dynamic phase, we present three clinical cases, evaluated at the Pediatric Pneumology Unit IRCCS G. Gaslini, Genoa, Italy.

**Figure 3:**
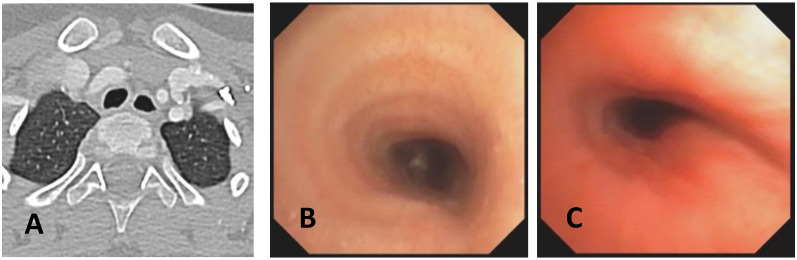
(**A**) Chest CT with CM: Tracheal lumen only slightly deformed at the junction with the IA. (**B**) SVBS: Shows only slight extrinsic compression at the junction of the IA. (**C**) DVBS: When the patient attempts to cough, the hypermobility of the pars membranacea appears, with subtotal occlusion of the tracheal lumen at the level of even slight extrinsic compression. **Abbreviations.** CT: Computed Tomography; CM: Contrast Medium; IA: Innominate Artery; SVBS: Static Videobronchoscopy; DVBS: Dynamic Videobronchoscopy.

**CLINICAL CASE 1**: A boy, 5 years old, with recurrent biphasic cough, both during infectious events and from physical exertion. The patient was evaluated with a Chest CT with CM, SVBS, and DVBS, and SVBS confirms only a slight extrinsic compression at the junction of the IA during quiet breathing. DVBS demonstrates how the tracheal lumen decreases significantly during coughing, mainly due to the pars membranacea hypermobility, without achieving complete closure; see Figure 3.

The patient was discharged with the following background medical therapy: inhaled corticosteroid and respiratory physiotherapy with a Positive Expiratory Pressure (PEP) mask, with a clinical follow-up after 6 months.

**Figure 4:**
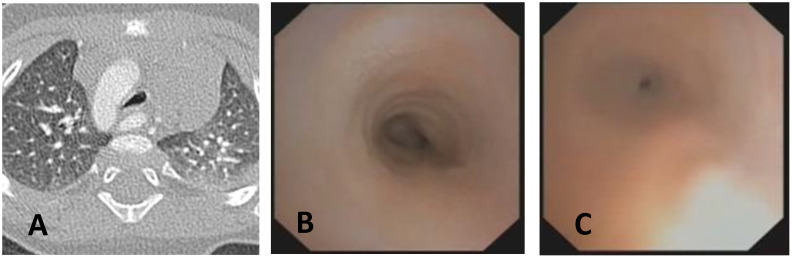
(**A**) Chest CT with CM: right aortic arch with lusory subclavian artery and marked deformation of tracheal lumen at this level. (**B**) SVBS: a patent tracheal lumen is present up to the junction between the middle and distal third tracheal lumen, where pulsating extrinsic compression appears on the anterolateral right wall, deforming and narrowing the tracheal lumen. The carina is patent, but the main right bronchus inlet is slightly deformed by the aortic arch. (**C**) DVBS, stimulating cough reflex: Almost complete occlusion of trachea. **Abbreviations.** CT: Computed Tomography; CM: Contrast Medium; SVBS: Static Videobronchoscopy; DVBS: Dynamic Videobronchoscopy.

**CLINICAL CASE 2**: A girl, 8 months old, with a prenatal diagnosis of right aortic arch with lusory subclavian artery, confirmed at birth by an echocardiogram. She had an absence of respiratory or gastrointestinal symptoms, with good growth. We performed Chest CT with CM, SVBS, and DVBS. The CT scan confirmed a right aortic arch with a lusory subclavian artery, causing marked compression/deformation of the tracheal lumen at this level, confirmed by SVBS; there was a narrowing of the tracheal lumen by more than 50% compared to the upper area. At this level, the DVBS demonstrated almost complete occlusion of the tracheal lumen under abdominal straining; see Figure 4.

Cardiac surgery followed TT discussion: The section of atretic left arch, resection of Kommerell’s diverticulum with reimplantation of left lusory subclavian artery on the ipsilateral carotid artery. SVBS one year after surgery: Well-preserved tracheal lumen up to the distal third, where only a slight pulsating deformity on the anterolateral right wall at the site of the aortic arch was still present. DVBS: Even when the patient performs abdominal straining, the occlusion of the tracheal lumen was no longer present. Endoscopic images were not presented.

**Figure 5:**
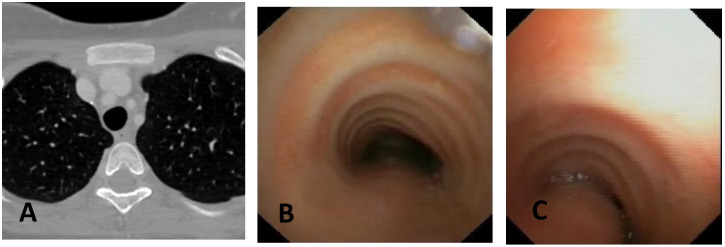
(**A**) Chest CT with CM: Slight tracheal compression from IA. (**B**) SVBS: Well-preserved tracheal lumen up to junction between the middle and distal third, where clear pulsating extrinsic compression from IA deforms the trachea. (**C**) DVBS: when the patient performs abdominal straining, the hypermobile pars membranacea completely occludes the trachea at the level of extrinsic compression. The presence of granulomas at this level proves this contact occurs recurrently. **Abbreviations.** CT: Computed Tomography; CM: Contrast Medium; SVBS: Static Videobronchoscopy; IA: Innominate Artery; DVBS: Dynamic Videobronchoscopy.

**CLINICAL CASE 3**: A girl, 15 years old, with a clinical history of biphasic cough and recurrent respiratory infections. She was already diagnosed with asthma and treated with different inhalation therapies without clinical benefit. The patient was evaluated with a Chest CT with CM and SVBS/DVBS. Both CT scan and SVBS showed slight tracheal compression at the junction of IA with the anterior tracheal wall; the lumen was deformed but not restricted. The DVBS demonstrates pars membranacea hypermobility during abdominal straining, protruding pathologically into the tracheal lumen, which was totally occluded at the level of IA compression. See Figure 5.

**Figure 6:**
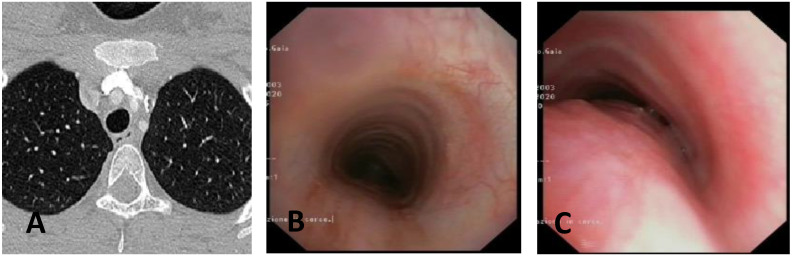
(**A**) Chest CT with CM: Well-preserved tracheal lumen even at the junction with the IA. Figure (**B**) SVBS: Persistent reduction in the lumen by 25–30% between the middle and distal third of the trachea, with no more visible transmitted pulsations and granulomas. (**C**) DVBS: Marked protrusion of the pars membranacea with abdominal straining, occluding the lumen by about 80–90%. **Abbreviations.** CT: Computed Tomography; CM: Contrast Medium; OAA: Open Anterior Aortopexy; IA: Innominate Artery; SVBS: Static Videobronchoscopy; DVBS: Dynamic Videobronchoscopy.

After collegial discussion with the TT, OAA was performed (see “Surgical Treatments” later). At the clinical follow-up one year later, infectious episodes were less frequent, but biphasic cough attacks were still present. The patient was again evaluated with Chest CT with CM and SVBS/DVBS. The chest TC and the SVBS showed a good outcome of the OAA procedure: well-preserved lumen along the entire tracheal length, and granulomas were no longer visible on the posterior tracheal wall. DVBS visualized persistence of marked protrusion of the pars membranacea, which occludes the lumen by about 80–90%, with abdominal straining. See Figure 6.

**Figure 7:**
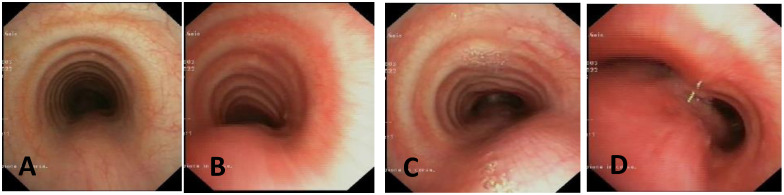
(**A**,**B**) SVBS: Well-preserved tracheal lumen, only moderately deformed at the site of previous extrinsic compression. (**C**,**D**) DVBS: Physiological protrusion of the pars membranacea into the tracheal lumen, which remains well patent even under abdominal straining (slight bulging only above the carina). **Abbreviations.** SVBS: Static Videobronchoscopy; DVBS: Dynamic Videobronchoscopy.

PT was performed (see “Surgical Treatments” later) after discussion with the TT. The follow-up was 6 months after the surgery: significant clinical improvement was observed with the disappearance of the biphasic cough. An endoscopic procedure was repeated: DVBS showed a well-opened tracheal lumen even under abdominal straining, with physiological protrusion of the pars membranacea into the tracheal lumen. See Figure 7.

These three clinical cases demonstrate the importance of performing, in suspected TM, both SVBS and DVBS to highlight changes in the tracheal lumen when intrathoracic pressure increases, such as during abdominal straining. These changes are not visible during spontaneous breathing but are responsible for the clinical symptoms presented by the patient in everyday life.

### 3.4. Indications for Surgical Treatment

**Figure 8:**
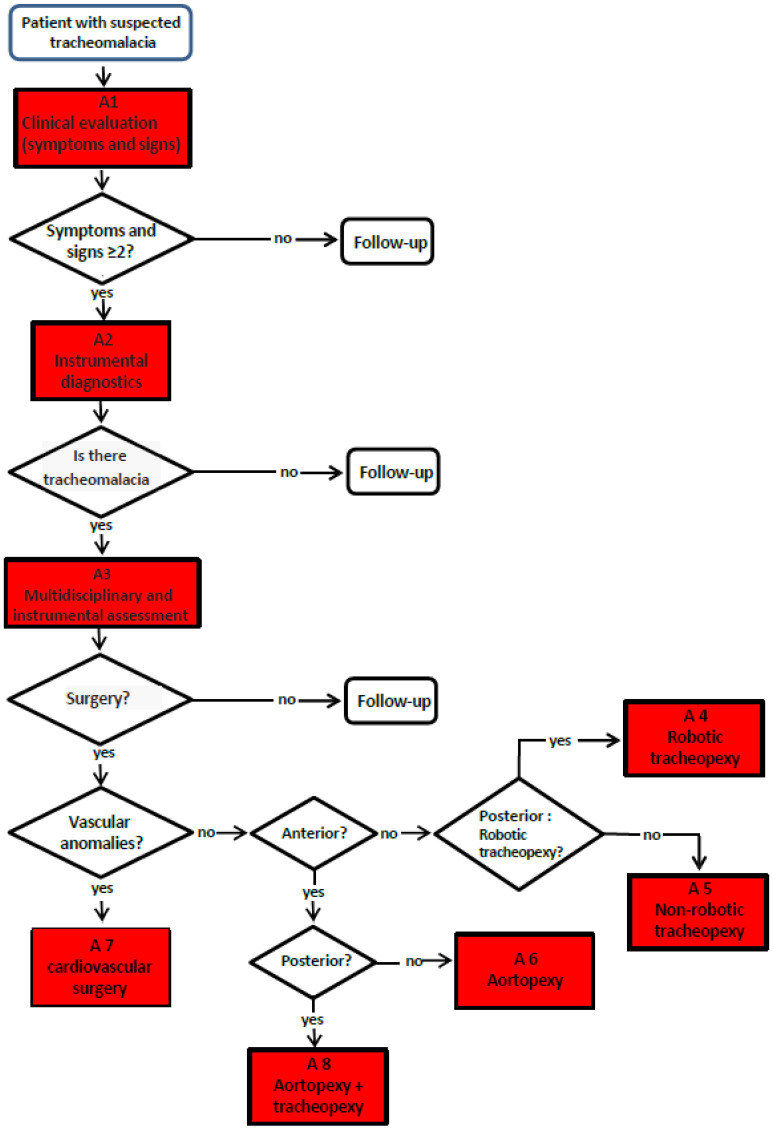
Flow chart for the management of patients with TM.

Primary TM (rarer), due to developmental alteration of the organ, or secondary TM (more common), due to extrinsic compression, mostly vascular, is the most common congenital tracheal anomaly, affecting about 1 in 2000 children [1]. Despite its relative frequency, the management of patients with TM varies greatly from center to center, lacking a clear decision-making algorithm to guide the clinician from diagnosis to treatment [29]. Within the Gaslini Institute TT, composed of pulmonologists, thoracic surgeons, cardiothoracic surgeons, otolaryngologists, radiologists, gastroenterologists, and anesthesiologists, we discuss all TM clinical cases in the weekly team meetings, according to this flow chart (Figure 8):

Although a flow chart for the management of patients with TM has been broadly established, there is still no standardization of the following:

4. The degree of clinical severity: biphasic cough, stridor, wheezing, recurrent respiratory infections, dyspnea, and respiratory failure.
5. The degree of TM severity according to radiological imaging (Chest CT with CM) and endoscopic criteria: S/DVBS.

Within the Gaslini Institute TT, we felt the need to develop a Tracheomalacia Score (TMS) that takes into account both of the following clinical symptoms: Clinical Score and endoscopic data. The Endoscopic Score helps choose the best therapeutic option for each TM patient: (1) vigilant waiting with medical therapy and respiratory physiotherapy, (2) AA, or (3) PT.

- **TM Clinical Score (TMCS)** was formulated in accordance with the experience of Gaslini TT, characterized by the absence, with a **score** = **0**, or presence, with a **score** = **1**, of certain symptoms (each symptom contributes with its score to the final sum score), stratified by age groups. Symptoms must be present even in substantial well-being, outside of acute infectious events.

**0–2 years**

☐Apparent Life-Threatening Event (ALTE);☐Biphasic or “choked” cough or abnormal tone;☐Inspiratory or expiratory stridor;☐Jugular or intercostal retractions;☐Recurrent pneumonia (>2 episodes) in a non-immunodeficient patient.

**2–6 years**

☐Recurrent/persistent biphasic cough; ☐Inspiratory or expiratory stridor with or without jugular or intercostal retractions; ☐Recurrent and prolonged lower respiratory tract infections (>6/year) in a non-immunodeficient patient; ☐Poor resistance to play; ☐Dysphagia or vomiting.

**>6 years**

☐Recurrent/persistent biphasic cough; ☐Recurrent and prolonged lower respiratory tract infections (>6/year) in a non-immunodeficient patient;☐Poor resistance to physical activity/sports; ☐Exertional cough, in the absence of bronchospasm; ☐Dysphagia or vomiting/gastroesophageal reflux.


In cooperative patients who can perform spirometry with a correct F/VC, a score of 1 is assigned if the expiratory phase of the curve shows a box or plateau appearance (see above: Section 3.2. Spirometry).

**Figure 9:**
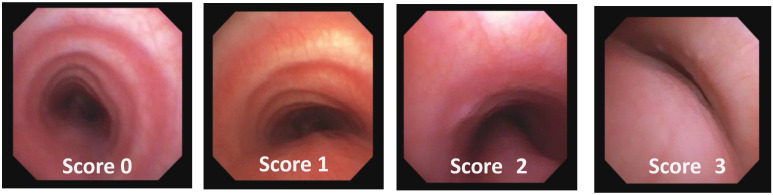
TMES Score 0–3, following the indications of the 2019 ERS statement on TM in children. **Abbreviations.** TMES: Tracheomalacia Endoscopic Score; TM: Tracheomalacia.

In our institute, each patient with suspected TM is evaluated according to this TMCS and, if at least two signs/symptoms are present, undergoes S/DVBS to formulate the following:

- **TM Endoscopic Score (TMES)** tracks the indications of the 2019 ERS statement on TBM in children [1]. The S/DVBS leads to the assignment of the following scores:

**Score: 0**Pulsating extrinsic deformation on the anterior tracheal wall, reduction in the APD of the trachea of less than 50% compared to the suprastenotic tract, even during expiration. Good representation of the cartilaginous rings. Figure 9, score 0 **Score: 1**Reduction in the tracheal APD between 50% and 75% compared to the suprastenotic tract, with an absence of contact between the anterior tracheal walls and the pars membranacea, even when the patient performs abdominal straining. Good representation of the cartilaginous rings. Figure 9, Score 1**Score: 2**Reduction in the APD of the trachea between 75% and 90% compared to the suprastenotic tract and/or anterior tracheal wall and pars membranacea, tending to touch, without complete closure of the lumen, even when the patient performs abdominal straining, with poor representation of the cartilaginous rings. Figure 1, score 2**Score: 3**Reduction in the APD of the trachea is already greater than 90% during the expiratory phase, when the tracheal lumen completely closes. Figure 9, score 3

The TMS is obtained by summing the TMCS and the TMES: if the total score is greater than 5, a surgical approach is indicated; otherwise, only medical therapy and clinical follow-up are recommended.

The development of a third score, the TM radiological score (TMRS), is currently in progress through a retrospective observational study (resulting from the collaboration between pulmonologists, radiologists, and surgeons) with the aim of correlating Chest CT imaging (with evaluation of specific markers) with the clinical and endoscopic data of patients with TM.

**Discussion**. The flow chart for the management of the patients with TM was shared and approved in its lines. The main focus was on the importance of DVBS to visualize how the tracheal lumen behaves when the intrathoracic pressure increases, when the patient tries to cough. Not all pediatric pulmonology centers directly perform bronchoscopy: the procedure is often performed by otolaryngologists or anesthesiologists, who rarely perform the dynamic phase of endoscopy. All pediatric pulmonologists agreed on the importance of DVBS and on the need to directly perform the procedure to obtain the best data on which to make surgical indications, and specifically whether anterior aortopexy or posterior tracheopexy. The need to develop a Tracheomalacia Score (TMS), as proposed by the Pneumology and Thoracic Surgery Unit Gaslini Children Hospital, was shared by most of the meeting participants. A verbal commitment to try to implement this tool was expressed, but a lot of work still needs to be done to have a TMS used in various Italian pediatric pulmonology centers to characterize TM patients.

## 4. Surgical Treatments

### 4.1. Introduction

The choice of treatment for TM depends on the etiology and severity of TM itself, the general and respiratory clinical conditions of the patient, and the presence of any comorbidities. Mild and moderate TM can be managed pharmacologically or non-invasively because, in most cases, there is an improvement in clinical symptoms as the patient grows. For the treatment of severe TM, a surgical approach is generally indicated, especially when there is a compromised respiratory function requiring mechanical ventilation, feeding difficulties, recurrent episodes of bronchopneumonia or PBB, and, every time, the barking cough is so frequent that it severely impairs the patient’s quality of life [1,30].

The main surgical options for the treatment of TM are (1) anterior aortopexy (AA), (2) posterior tracheopexy (PT), and, more rarely, (3) tracheal resection. The use of (4) resorbable endotracheal/bronchial stents, placed endoscopically, is also increasingly common, particularly in cases of surgical failure or when surgery is contraindicated.

The first treatment of TM in most centers is usually medical, according to local experience/preferences. In non-responders or in severe cases, a surgical approach may be considered; we summarize the most common surgical treatments for TM, focusing on the different approaches and the technical aspects of each technique. This is a multicenter symposium, and each speaker describes the preferred approach and technique performed in their center.

### 4.2. Open Anterior Aortopexy (OAA)

OAA (via right or left anterior thoracotomy, or sternotomy) has always been considered the standard procedure for the treatment of TM due to anterior tracheal vascular compression by an anomalous course of the IA [31,32]. At the Cardiosurgery Division of IRCCS G. Gaslini, Genoa, Italy, from June 2011 to December 2023, 131 patients with symptomatic tracheal vascular compression due to an anomalous course of the IA underwent OAA.

Patient population

- A total of 131 patients;
- Age: 5.1 ± 4.2 years (range 0.2–16.39 years);
- Female: 32%, Male: 68%;
- TEF: 38 patients (29%);
- Comorbidities: cardiopathy, chromosomal abnormalities, autism, etc.: 37 patients (28.2%);
- A total of 107 patients (82%): Upper ministernotomy (split sternum);
- A total of 24 patients (18%): Right or left anterior thoracotomy.

Additionally, six patients with left main stem bronchus malacia underwent section and suture of the arterial ligament and posterior aortopexy (original approach) [33].

The right or left thoracotomy approach was used in the early years of the reported experience. Currently, ministernotomy is considered the preferred access route for OAA. With the patient in the supine position, the sternum is split through a 2–3 cm skin incision. Subtotal thymectomy is performed to create sufficient space in the anterior mediastinum for AA. The pericardium is opened, and two 4-0 Prolene sutures mounted on pledgets are usually placed on the aorta adventitial tunic for traction: one at the base of the IA origin, the other after the origin of the left carotid artery, in the concavity of the arch. Under endoscopic control, the sutures are first anchored to the sternum, transfixing it, and then tied on the anterior sternal table. The chest is closed with steel wires, and a 10 Fr pericardial drain is placed under suction. The pleurae are drained only in case of accidental opening. The procedure is carried out in just 30–40 min, with the patients being extubated in the operating room if their weight is >10 kg, and after a two-hour observation period if they weigh <10 kg. Average postoperative stay: 8 ± 7 days (range 4–50 days)

With zero mortality, the rate of major complications is very low: less than 6%. The average hospital stay is short: about a week.

Complications

- Mortality: zero;
- One major bleeding;
- Two pericardial effusions (percutaneous drainage);
- Three cases of chronic pericarditis;
- Two transient peripheral nerve injuries (phrenic nerve).

Complete follow-up was performed on 109 patients, typically seeing the patient 8–12 months later, with S/DVBS. CT with CM is reserved for still-symptomatic cases or if SVBS shows persistent pulsatile extrinsic compression with residual TM.

Follow-up

- A total of 109/131 patients (83%);
- Average follow-up months: 29.75 ± 27.17 (range 3.3–130.66).

Clinical Outcome

- A total of 52% were asymptomatic;
- A total of 35% were mildly symptomatic;
- 1A total of 3% were still symptomatic.

Endoscopic Outcome: 8–12 months after OAA

- Increase in tracheal APD and disappearance of pulsatility: 85%;
- Unchanged diameters and pulsatility: 15%;
- Requiring second-stage treatment with PT: 7 patients (6.4%).

A total of 87% of operated patients report clinical improvement, with 52% completely asymptomatic and 35% significantly improved; the VBS data generally correspond with clinical outcome. In cases of persistent TM, especially if DVBS shows a reduction in tracheal APD due to hypermobility of the membranous part, with no clinical improvement, our protocol includes PT via right thoracoscopy.

In conclusion, TM, due to vascular compression by IA not being so rare, requires a multidisciplinary approach by a dedicated TT; it is treatable with excellent results with OAA, ensuring excellent short- and long-term outcomes.

### 4.3. Thoracoscopic Anterior Aortopexy (TAA)

At the Pediatric Surgery Division of Grande Ospedale Metropolitano, Milano, Italy (GOM Hospital), the experience with the surgical correction of TM using the TAA technique began in 2023. The first case was a male newborn, operated on at 2 days of life (GA 35 + 6, BW 1490 g) for type III EA, who, in the weeks following the surgery, had repeated episodes of respiratory arrest requiring prompt resuscitation. VBS in spontaneous breathing and Chest CT with CM showed severe TM without vascular rings or compressions. The indication for AA was based on literature reporting satisfactory efficacy rates of the procedure and its feasibility even with minimally invasive surgery (MIS) thoracoscopic technique [34,35]. The advantages of the thoracoscopic surgical approach over thoracotomy in infancy and childhood are demonstrated in terms of reduced postoperative pain, hospitalization and functional recovery times, long-term musculoskeletal complications, and cosmetic aspects [36,37,38,39,40]. Despite favorable experiences already being reported [41], recent studies have shown that some results of MIS, especially regarding recurrence, appear inferior to those of traditional surgery, specifically OAA [42]. Therefore, a systematic review was conducted according to PRISMA guidelines to clarify unclear aspects of this comparison. Intraoperative complications and mortality were comparable between the two groups, while the recurrence rate was higher in the MIS group. Detailed analysis of the MIS approach revealed that the traditional technique was often not followed, not performing pericardiotomy, and not using Teflon pledgets. Conversely, thoracoscopic experiences faithfully reproducing traditional steps, including pericardiotomy and the use of pledgets, showed results comparable to the traditional technique. Before establishing a definitive surgical approach, preoperative CT with CM is essential in determining the presence and thickness of the thymus to evaluate the recoverable anterior space for traction after thymectomy. It also allows precise assessment of the extent of malacia and consequently choosing the most appropriate approach: AA vs. PT. Preoperative CT with CM. This also allows preoperative planning of the correct positioning of trans-sternal sutures to achieve the most effective traction.

In light of all these observations, it was decided to proceed with the MIS approach. Anesthetic management included the ipsilateral lung exclusion with an endobronchial blocker, Near-Infrared Spectroscopy (NIRS), and Sedline monitoring. The cardiac surgeon and the table for urgent sternotomy conversion were always available in the operating room.

The surgical technique steps at our center are as follows:

- Preliminary VBS;
- Supine position with left flank and shoulder elevated by 30°;
- Three trocars: 3 mm for patients <1 year; 5 mm for children >1 year;
- Opening of the mediastinal pleura;
- Thymectomy (possibly total);
- Isolation and lifting of the IA;
- Pericardiotomy;
- Non-absorbable sutures with Dacron pledgets on the aorta and IA;
- Pericardial flap;
- Retrieval of trans sternal sutures with “Suture Passer”;
- Retrosternal scarification;
- Tension and closure of the sutures subcutaneously;
- No drainage;
- VBS postoperative control.

In the following 12 months, another six patients underwent TAA, with a median age of 3 years (6 months–6 years) and a median weight of 15 kg (3–23 kg). All patients were awakened at the end of the procedure, without the need for intensive care admission. No patient had a thoracic drain placed. Feeding was resumed on the same day of the surgery, and discharge was relatively early (median fourth postoperative day, range 3–21 days). All showed clinical improvement: complete in five and partial in two, who subsequently underwent thoracoscopic surgical revision.

The literature data [43] show that the general frequency of complications for AA is as follows:

- Pneumothorax and/or pleural effusion: 3%;
- Pulmonary atelectasis: 2.5%;
- Pericardial effusion: 2%;
- Phrenic nerve paralysis: 1.3%;
- Hemorrhage: 1%;
- Recurrence: 1%;
- Death: 6%.

In our series, we had only one case of bleeding from the IA, effectively managed through conversion to ministernotomy and completion of the procedure via open approach.

Based on the results of our preliminary experience, the MIS approach to AA appears feasible and safe, and it can therefore assume a role of choice, if some essential prerequisites are guaranteed: an adequate (certified) learning curve in pediatric thoracoscopy, dedicated anesthetists, a surgical team experienced in open and MIS thoracic surgery, a pediatric cardiac surgeon at the operating table, an accessory operating table for urgent sternotomy/thoracotomy conversion, and constant postoperative review of individual cases treated for critical analysis. These procedures should be considered the prerogative of a multidisciplinary pediatric TT.

### 4.4. Posterior Tracheopexy (PT)

PT consists of fixing the posterior wall of the trachea (pars membranacea) to the anterior longitudinal ligament of the spine using sutures [44]. DVBS, when documenting the hypermobility of the pars membranacea, occluding almost completely the tracheal lumen during abdominal straining, is the gold standard to indicate this surgical procedure. Endoscopic images also indicate the extent of the posterior tracheal wall that shows hypermobility and thus the tracheal segment to be fixed to the spine.

In the period 2018–2024, 18 patients underwent PT at UOSD Thoracic and Airway Surgery, IRCCS G. Gaslini, Genoa. The “classic” surgical approach was right posterolateral thoracotomy, while sternotomy was preferred only in specific cases. Recently, the surgical procedure has been increasingly performed using minimally invasive techniques: both Video-Assisted Thoracoscopic Surgery (VATS) and Robotic-Assisted Thoracoscopic Surgery (RATS) [25,26]. After mobilizing and laterally displacing the esophagus and thoracic duct, non-absorbable sutures are passed between the posterior membrane of the trachea, without crossing it, and the anterior longitudinal ligament of the spine under VBS control. In this way, the pars membranacea is fixed to the spine, resulting in a significant reduction in its mobility during expiration, limiting its protrusion into the tracheal lumen when the patient coughs, ensuring good preservation of the tracheal lumen and the disappearance of the biphasic cough.

Patient population

- A total of 18 patients: 11 males, 7 females;
- Average age: 10.50 ± 5.57 years (range: 3.33–22.89 years);
- A total of 6 patients (33.3%): EA/TEF;
- A total of 1 patient: congenital diaphragmatic hernia (CDH);
- A total of 6 patients (33.3%) had previously undergone AA;
- A total of 1 patient: AA was performed simultaneously with the PT procedure;
- Average postoperative hospital stay for patients undergoing RATS was 8 ± 11 days (range: 3–16 days);
- Average follow-up of 10.23 ± 11.62 months (range: 1.41–40.62 months).

Detailed analysis of operative times revealed considerable variability between different approaches, with operative times ranging from 105 to 320 min and console times for RATS ranging from 75 to 215 min.

Intraoperative complications were recorded only in one patient, related to the AA performed simultaneously; no intraoperative complications occurred in patients treated with RATS. Postoperative complications affected 4 out of 18 patients (22%), of which 4 (28.6%) were treated with RATS, all with favorable outcomes with conservative treatment.

Postoperative complications in 4 out of 18 patients (22%) are as follows:

- Two patients: thoracic duct injury;
- One patient: dysphagia;
- One patient: esophageal perforation, treated with stent placement.

The presence of comorbidities such as esophageal malformations and congenital heart diseases influenced both the choice of surgical approach and the postoperative outcomes, indicating the need for a personalized operative plan for each patient.

The Clinical outcome, evaluated in 16 of the 18 patients, are as follows:

- Complete resolution of respiratory symptoms in 50% of cases (8/16);
- Clinical improvement in 43.75% (7/16);
- Stability in 6.25% (1/16);
- Overall benefit in 93.75%.

In conclusion, in cases of hypermobility of the pars membranacea, PT, especially with the use of minimally invasive techniques such as RATS, represents an effective solution with manageable complications and a high rate of clinical success.

**Discussion**: There was a general consensus in underlining the importance of the dynamic phase of bronchoscopy for differentiating patients who require aortopexy or tracheopexy, and interventions are now well described in their procedural steps. In the absence of a TMS shared by the various centers, no unity of views emerged, both on the degree of tracheomalacia that could justify the surgical indication and also, above all, on the timing of the operation. If the most severe cases of TM are generally treated surgically within the first 2 years of life and there are no choices other than surgical treatment, there would be a lower concordance of indications regarding the vast majority of patients with TM but with less severe clinical symptoms. There was a good consensus to indicate surgery in cases of TM associated with a diagnosis of protracted bacterial bronchitis, especially in cases with poor response to medical therapy. Surgical treatment of TM in these patients was judged to be important in hindering the progression to the development of bronchiectasis.

## 5. The Patient with Esophageal Atresia (EA) with/Without Tracheoesophageal Fistula (TEF)

### 5.1. The Severe TM in Patients with EA with/Without TEF

EA, with or without TEF, is a congenital malformation characterized by the interruption of the continuity of the esophagus and an abnormal communication: a fistula, between the digestive and respiratory tracts, also known as TEF. The morphogenesis of this anomaly occurs in the early stages of embryonic development (fifth week of gestation), when the “foregut” forms the tracheoesophageal septum, which divides a dorsal portion (esophagus) from a ventral portion (trachea) [45]. EA is often associated with other malformative anomalies: VACTERL, CHARGE syndrome, and cardiac or genitourinary defects. This association highlights a common genetic defect that alters the early stages of embryonic development [46].

TEF occurs when there is an alteration in the physiological process of detachment of the airway from the foregut (the primitive esophagus) so that an abnormal communication between the esophagus and the trachea is maintained. The presence of TEF interferes with the regular differentiation of the tracheal wall, which, at this level, presents poorly formed cartilaginous arches, resulting in the most severe form of primary TM, because the malacia is intrinsic to the tracheal wall. This condition, typical of patients with TEF, is generally also associated with secondary or extrinsic TM due to the compression from the IA, which greatly contributes to further worsening the patency of the tracheal lumen [47]. TM in these patients is always present and accompanied by severe respiratory symptoms, as a vicious circle is established: the impairment of mucociliary clearance causes secretion stagnation, which favors persistent bacterial colonization with the development of PBB and pneumonia, leading to the final formation of bronchiectasis [48,49]. Patients with EA/TEF present therefore recurrent respiratory symptoms during childhood, adolescence, and adulthood, which require long-term pulmonary follow-up.

**Figure 10:**
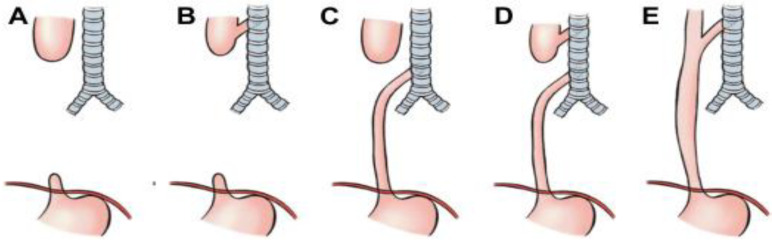
The Gross classification of EA, based on the presence and location of TEF. **Type** (**A**) EA without fistula; **Type** (**B**): EA with TEF between the upper segment and the trachea; **Type** (**C**) EA with TEF between the lower segment and the trachea (most common type: 84% of cases); **Type** (**D**): EA with 2 TEFs connecting both segments to the trachea; **Type** (**E**) TEF without EA (H-type fistula). **Abbreviations.** EA: Esophageal Atresia; TEF: Tracheoesophageal Fistula.

EA, with or without TEF, has an incidence of about 1 in 3500/4500 live births, and five types are recognized [50,51], according to the Gross classification of EA, based on the presence and location of TEF: Figure 10.

**Figure 11:**
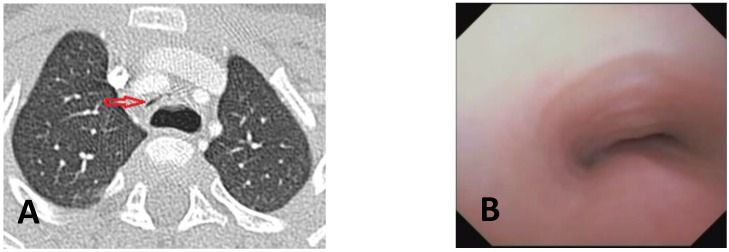
(**A**) Chest CT with CM: Tracheal lumen completely collapsed (arrow) at the level of anterior compression by the IA and posteriorly by the dilated esophagus. (**B**) SVBS: Endoscopic view of the trachea above the TEF: Tracheal lumen almost completely collapsed even during quiet breathing. **Abbreviations.** CT: Computed Tomography; CM: Contrast Medium; IA: Innominate Artery; SVBS: Static Videobronchoscopy.

**CLINICAL CASE**: A baby was born prematurely (32 + 4 weeks), and the pregnancy was complicated by polyhydramnios. At birth, respiratory distress required CPAP support. Due to excessive salivation and difficulty advancing the nasogastric tube (NGT), an EG-EF was performed; it showed dilation of the proximal esophagus ending in a blind pouch, suggestive of EA, marked gaseous distension of the intestinal loops, indicative of a possible distal TEF, confirmed by bronchoscopy: EA type C. On the first day of life, ligation of the TEF and end-to-end esophageal anastomosis showed good results. In the first year of life, recurrent wheezing occurred with frequent emergency room visits, with one hospitalization for bronchiolitis requiring O2 support by High Flow Nasal Cannula (HFNC), also with a persistent choking cough. Diagnostic tests included Chest CT with CM and SVBS, performed at around 13 months of age, showing a completely collapsed tracheal lumen, anteriorly by IA compression and posteriorly by the dilated esophagus. See Figure 11.

**Figure 12:**
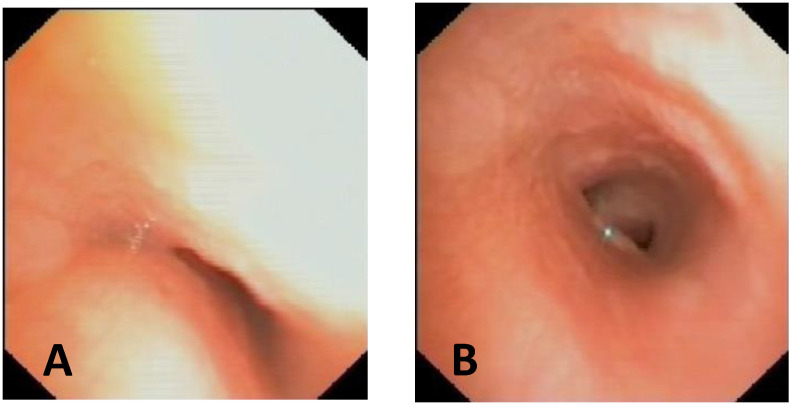
Intraoperative VBS during OAA (after thymectomy): (**A**) Without traction on the aortic arch and (**B**) with a significant increase in tracheal lumen while traction is exerted on the aortic arch. **Abbreviations.** VBV: Videobronchoscopy; OAA: Open Anterior Aortopexy.

OAA was performed via ministernotomy at 16 months of age. Intraoperative VBS, performed after thymectomy, showed a satisfactory opening of the tracheal lumen, particularly significant while traction was exerted on the aortic arch. See Figure 12.

**Figure 13:**
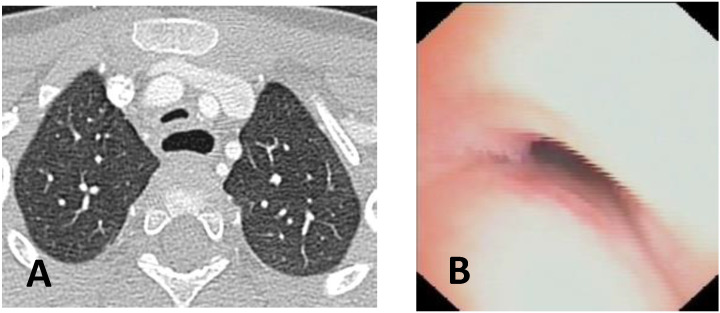
(**A**) Chest CT with CM: the IA appears well pulled anteriorly towards the sternum, but a severe TM persists with >75% occlusion of tracheal lumen, also related to the presence of a large esophagus compressing the tracheal lumen from behind. (**B**) The SVBS confirms the persistence of TM, with a patent but severely deformed tracheal lumen even in quit breathing. **Abbreviations.** CT: Computed Tomography; CM: Contrast Medium; IA: Innominate Artery; TM: Tracheomalacia; SVBS: Static Videobronchoscopy.

Despite the surgery, with favorable intraoperative control, the patient continued to experience recurrent cough, frequent need for antibiotic therapy cycles, repeated emergency room visits, and four hospitalizations for asthmatic bronchitis requiring HFNC. At 3 and a half years old (2 years after the OAA) the patient was reevaluated by Chest CT with CM and SVBS. Both exams showed that, although the IA appeared well pulled anteriorly towards the sternum, a severe TM was persistent with >75% occlusion of tracheal lumen. See Figure 13.

**Figure 14:**
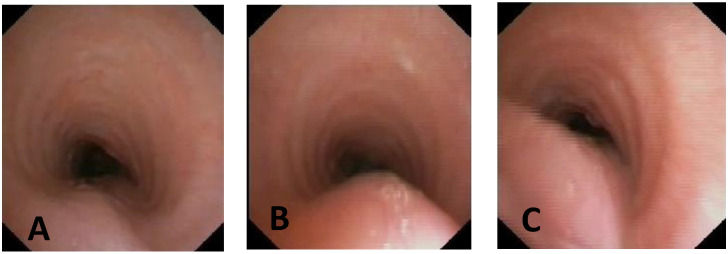
(**A**) SVBS: Good patency of the tracheal lumen; (**B**,**C**) DVBS: The lumen maintains a good patency even when the patient is performing abdominal straining, causing an increase in intrathoracic pressure. **Abbreviations.** SVBS: Static Videobronchoscopy; DVBS: Dynamic Videobronchoscopy.

After collegial discussion within the TT, a PT (see surgical procedures) was planned, which involves the partial displacement of the esophagus laterally to the right. After the surgery, there was a prompt clinical improvement with the disappearance of the recurrent biphasic cough. SVBS and DVBS, performed 6 months after the PT, showed a good patency of the tracheal lumen with almost complete restoration of the “bridge arch” structure of the wall, and a good lumen patency even when the patient was performing abdominal straining in an attempt to cough. See Figure 14.

In the clinical follow-up post-PT (the patient is now 7 years old), no more hospitalizations or antibiotic cycles were needed.

In conclusion, this clinical case highlights how, especially in patients with EA and TEF, AA may not be resolutive, making PT necessary for significantly improving the patient’s quality of life.

### 5.2. Indications for Consensual PT During Esophageal Recanalization in EA Patients

The execution of PT requires lateral displacement of the esophagus, followed by fixing the membranous part of the trachea to the anterior longitudinal ligament. This surgical step is unnecessary during the correction of EA because the esophageal segments are far apart, leaving the posterior tracheal wall exposed. Despite this apparent technical advantage, until 2017, there was no literature describing the execution of PT concurrent with EA treatment. The first to describe the technique in 2018 were colleagues from Utrecht: 4 patients were treated with the thoracoscopic technique [52], followed by the Aerodigestive Team from Boston with 18 patients, mostly treated with the open technique. Utrecht later described an additional 14 patients, and Boston, this year, described 21 patients, some also thoracoscopically treated [53], 17 patients were described in China [54]. These reports demonstrate that this procedure reduces respiratory morbidity and TM complications without increasing the complexity of the intervention and avoiding much riskier secondary procedures [53,54,55]. However, the short follow-up, heterogeneity, and complexity of the case series, sometimes approached with different techniques and all numerically very limited, necessitate the execution of prospective multicenter randomized studies [56,57].

**Figure 15:**
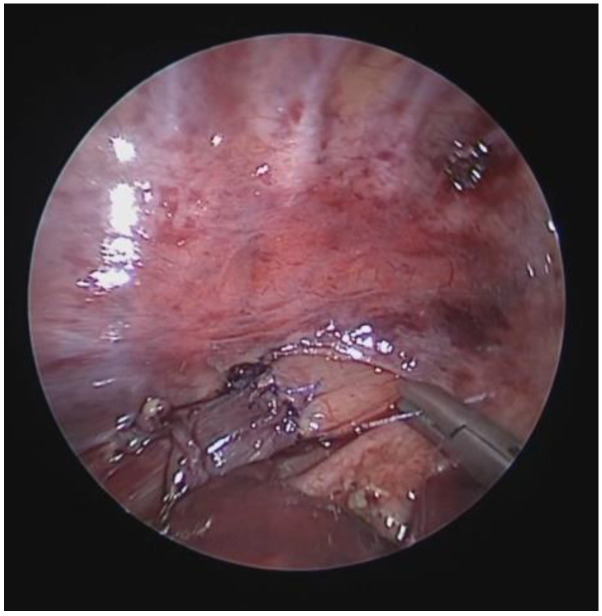
The distal esophageal segment projecting into the trachea as TEF is isolated and ligated with a transfixing suture and then sectioned.

In our center: Pediatric Surgery Unit, Ospedale Filippo Del Ponte, Varese, Italy, the procedure for correcting EA begins with VBS in a spontaneously breathing child, followed by rigid bronchoscopy. The surgery is then performed using a thoracoscopic technique, first isolating the proximal esophageal segment, then the distal segment projecting into the trachea as TEF. This is ligated with a transfixing suture and sectioned; the upper esophageal segment is then mobilized, freed from the posterior tracheal wall, opened, and anastomosed with its distal counterpart, so that the esophagus is canalized. See Figure 15.

**Figure 16:**
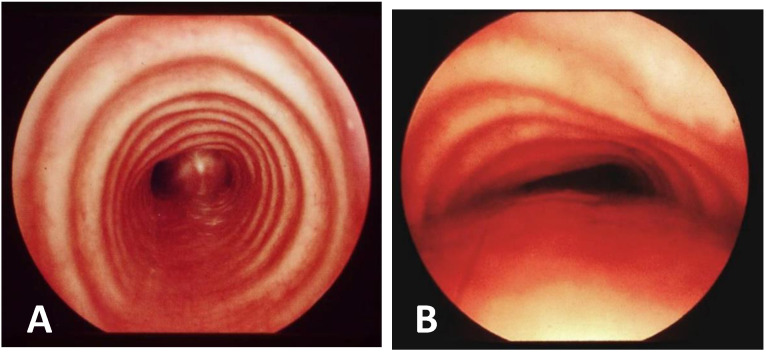
In the presence of a trachea like the one shown in (**A**), there are no indications of consensual PT, while the indications exist in the presence of the image in (**B**).

To answer the question of whether there are indications for PT in patients with EA and TEF, each patient must be evaluated individually, as shown in the two cases in Figure 16.

**Figure 17:**
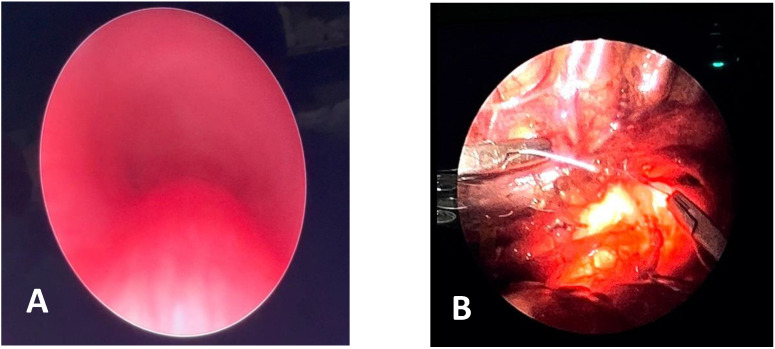
(**A**) Preoperative endoscopic image of a patient with EA and TEF, where there is TM. (**B**) The posterior wall of the trachea is therefore anchored (under endoscopic guidance), easily and without complication, to the anterior longitudinal ligament of the spine. **Abbreviations**. EA: Esophageal Atresia; TEF: Tracheal–Esophageal Fistula; TM: Tacheomalacia.

We recently encountered a patient with EA, and Figure 17A is the preoperative image (of poor quality) of their trachea. Well, we proceeded with the PT and, therefore, before performing the esophago–esophageal anastomosis, we anchored the posterior wall of the trachea (under endoscopic guidance) to the anterior longitudinal ligament of the spine, and the procedure was performed without difficulty and without complications; see Figure 17B.

In conclusion, in mild to severe cases of TM, the PT performed during the primary thoracoscopic EA correction can prevent potentially deleterious sequelae that may complicate the lives of EA patients [53]. Therefore, each patient must be carefully evaluated, and it is essential to always perform preoperative VBS. Additionally, having a team experienced in neonatal MIS is crucial for minimizing functional and aesthetic outcomes.

### 5.3. Endoscopic Treatment of Recurrent/Persistent TEF

Before the 1980s, the treatment of choice for recurrent or persistent TEF was classic open surgery. In the early 1980s, the first attempts at endoscopic obliteration of recurrent/persistent TEF after surgical closure were made, mainly due to the high complication rates following classic surgery [58]. Over the years, numerous strategies have been employed to achieve endoscopic closure of TEF, from the use of diathermocoagulation to simple mucosal denudation, to the abrasion of the edges plus the use of sealing agents, and even the placement of tracheal stents [59,60].

In our center, Otolaryngology Unit, IRCCS G. Gaslini Genova, Italy, endoscopic closure of TEF has been practiced since 2017, for a total of seven patients (two of whom are still in treatment). The patient age range is 4 months–4 years, with a median of 8 months. The weight of treated patients is 4.2–7.8 kg. With the use of a rigid bronchoscope, we first abrade the fistula edges with a CO_2_ laser fiber, then we locally apply a 50% Trichloroacetic Acid (TCA) solution. Each endoscopic session involves, after the laser, two applications of TCA, with an average total contact time with the mucosa of about 100 s. The average number of sessions required for closure was 3.6. Closure has been achieved so far in five out of seven cases (71%); the fifth and sixth cases are still in treatment, and three and four applications of TCA have been performed so far, respectively. No significant post-procedure complications have been reported. The following are our conclusions:

- Endoscopic treatment for the closure of recurrent TEF is reliable and has few complications.
- The method with laser edge abrasion, followed by two local applications of TCA, has proven to be the most effective. The application time of TCA to the abraded edges of the TEF is variable and operator-dependent; we maintain a total time (two applications) of about 100 s.
- Treatment limitations include patient weight (not less than 4 kg) and the possibility of using a rigid bronchoscope of at least 3.5 mm diameter.
- Repeated applications (an average of four in the literature) are necessary to achieve closure; we wait about 20 days between sessions.
- So far, no limit has been set beyond which the treatment is considered ineffective.

**Discussion**. There was a unanimous consensus in judging EA patients as the most severe TM cases, in which tracheoscopy should be performed early, if possible, simultaneously with surgical recanalization of the esophageal lumen. The consensual execution of posterior tracheopexy during esophageal recanalization is currently a practice that is still not very widespread among pediatric surgeons: to our knowledge, it is currently practiced in a few Italian pediatric centers, in selected cases of AE.

## 6. Follow-Up of the Patient with TM

Given the varying severity of clinical presentations and the heterogeneity of associated clinical conditions, the management of follow-up for patients with TM and TBM should aim for a personalized approach. As demonstrated by the ERS statement [1], numerous syndromes can be associated with the presence of TBM, which can represent either the primary clinical condition or a minor part of the patient’s spectrum of comorbidities (mostly neurological or cardiological).

Patients with TM require careful follow-up, especially for the recurrence of respiratory symptoms present from the early years of life: recurrent lower respiratory tract infections, wheezing, recurrent bronchitis, and pneumonia. These complications are particularly frequent in the most severe cases of TM, such as with patients with EA/TEF [61,62,63]. Regarding these patients, the ERNICA consensus statement [64] emphasizes the need for long-term longitudinal monitoring, based on a multidisciplinary program (surgical, gastroenterological and nutritional, and pulmonological) but managed by a single coordinating specialist, who can also decide on performing invasive procedures under sedation (e.g., esophagogastroduodenoscopy and VBS).

The ERNICA recommendations suggest that the longitudinal monitoring of these patients should start from hospital discharge and continue through the transition age and possibly into adulthood. Attention should be particularly focused not only on the proper management of recurrent infections but also on growth and weight gain, and the prevention of gastroesophageal reflux disease (GERD), particularly frequent in TEF patients. Additionally, it is mandatory to have a supportive and informative role from the pediatrician following the patient at home, both towards the family and the child affected by TBM.

The recurrence of respiratory symptoms is also a frequent issue in patients with intrathoracic vascular anomalies (complete or incomplete vascular rings), where the abnormal vessel often causes TM due to extrinsic compression. Although there is a clear surgical indication for some of these conditions, surgical treatment may not be followed by the immediate resolution of associated respiratory and/or digestive symptoms, as the TM takes time to improve. The persistence of symptoms up to a year after the correction of the anatomical anomaly is widely reported in the literature, especially in children with a double aortic arch, compared to children with a complete vascular ring from the right aortic arch [65].

Objectives of follow-up for patients with TM:

- Improvement of airway patency;
- Enhancement of mucociliary clearance;
- Prevention of recurrent respiratory infections and PBB;
- Reduction in the risk of lung damage;
- Improvement of long-term respiratory prognosis.

Diagnostic tools useful in respiratory follow-up, especially when periodic patient evaluation reveals a worsening of the respiratory condition, include the following [62,64]:

- VBS with bronchoalveolar lavage (BAL);
- Chest CT with or without CM;
- PFT: baseline and post-salbutamol;
- Exercise tests (walk test/cardiopulmonary exercise test);
- Nocturnal oximetry;
- Microbiological examination of sputum and/or BAL.

S/DVBS is certainly the gold standard for studying TBM, and during the examination, BAL can also be performed to check if the patient’s lungs are colonized by pathogenic germs. Chest CT with CM, and particularly Dynamic CT, has proven to be a valid alternative to VBS, having a good sensitivity and specificity in detecting and defining TBM [66]. Considering the rapid execution and the lack of need for deep sedation, it can be considered in fragile or clinically unstable patients, or every time the general sedation is contraindicated.

Baseline PFT may show a particular morphology of the expiratory phase of the F/VC with deflection/tendency to plateau in the expiratory phase not modifiable with bronchodilation, so the test can be used to assess the severity of TM in these patients [67].

The walk test or cardiopulmonary exercise test allows for objectifying effort dyspnea, detecting any variations in Saturation O_2_ values during imposed activity, and thus estimating functional capacity [68,69]. Nocturnal oximetry monitoring in patients with TM is indicated if obstructive apnea events secondary to tracheal wall collapse are suspected, or comorbidities that may cause apneic episodes are present. Due to the inefficient mucociliary clearance of the malacic tract, children with TM often experience infectious episodes of the lower respiratory tract: in such circumstances, microbiological monitoring of respiratory secretions (sputum or BAL) can be useful for managing a possible subsequent episode of respiratory exacerbation [70].

In light of the findings during the diagnostic process in the follow-up, the pulmonologist should focus the medical management of patients with TM on managing bronchial hyperreactivity and infectious episodes. The cornerstones of therapy are therefore as follows: (a) Maintenance inhaled steroid therapy in patients with bronchial hyperreactivity, and (b) broad-spectrum antibiotic therapy, to be started early in the presence of symptoms suggestive of respiratory exacerbation involving the lower airways. Currently, there are no studies on the usefulness of azithromycin prophylaxis in patients with TM: the indication for its use is extrapolated from data on patients with non-cystic fibrosis bronchiectasis and frequent respiratory exacerbations. (c) Respiratory physiotherapy utilizes devices promoting the mobilization of respiratory secretions (PEP mask or similar devices), and (d) immunoprophylaxis, where Respiratory Sincitial Virus vaccination can be administered in the first year of life in patients with tracheobronchial tree anomalies, as well as annual influenza vaccination [70].

In conclusion, in the absence of precise guidelines regarding the follow-up of patients with TBM, within a multidisciplinary approach to the patient, the pulmonologist should personalize the diagnostic-therapeutic pathway, based on the reported symptoms and clinical findings, especially in cases with other comorbidities.

**Discussion.** The follow-up of patients with TM, whether treated conservatively or surgically, was judged by all participants to be essential: the patients must be followed over time, even after a surgical procedure, with good clinical outcomes. It was emphasized that follow-up must always be multi-specialist, and possibly carried out at the center where the patient underwent the entire diagnostic and therapeutic process.

## 7. Respiratory Assistance in Patients with TM

### 7.1. The Tracheal Splint

As early as 1968, Vasko et al. described the treatment of TBM by external application of specially shaped costal cartilage [71]. In subsequent decades, synthetic material devices were introduced, particularly in the 1970s and 1980s, when Filler et al. [72] used supports made of silicone elastomers (Silastic), reinforced with polypropylene mesh (Marlex). Later, in 1997, Hagl et al. used devices made of polytetrafluoroethylene (PTFE). In the last 10 years, there has been an increase in studies on the use of external support devices for the tracheobronchial wall or splints; in 2017, Ando et al. published a series of 98 patients with TBM who underwent placement of PTFE tracheobronchial splints, with good results: low morbidity and mortality [72].

**Figure 18:**
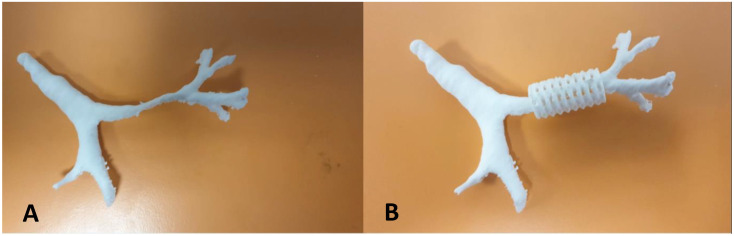
(**A**) A three-dimensional airway model; (**B**) an example of bronchial splint in polycaprolactone and hydroxyapatite on the airway model.

In 2015 and 2019, the Green group presented their experience with the implantation of tracheobronchial splints, produced using 3D printing, made of polycaprolactone (96%) and hydroxyapatite (4%) [73,74]. Patients underwent chest DCT and VBS to confirm diagnosis, position, and extent of TBM. From the CT images and three-dimensional reconstructions: Figure 18A, the length and diameter of the malacic segment were calculated for each patient, from which the 3D printing of the splint was performed, using a laser sintering machine (96% polycaprolactone/4% hydroxyapatite): Figure 18B.

**Figure 19:**
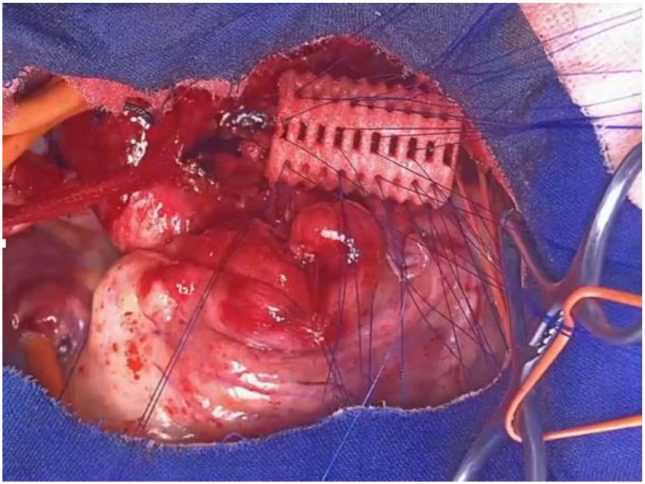
After 3D printing of the custom-made splint, Prolene sutures are placed on the tracheal or bronchial wall of the malacic segment. The sutures then pass through the splint slots, so that it is fixed to the wall, suspending the trachea or bronchus within it. The correct positioning of the splint and the achieved patency of the malacic segment are verified by intraoperative VBS.

Our recent experience at the Bambin Gesù Pediatric Hospital in Rome, with three patients (one tracheal splint and two bronchial splints), is based on the aforementioned indications from Green’s work for the production of the devices [73,74,75,76]. For each treated case, authorization was obtained from our ethics committee for “compassionate use” and from the Ministry of Health for the suitability of the device. Each device undergoes static and dynamic mechanical tests (compression, torsion, and breakage) to verify its elastic, resistance, and failure characteristics. The devices are sterilized using ethylene oxide. The composition of the splint is as follows: 96% polycaprolactone and 4% hydroxyapatite, which allows the maintenance of initial properties without significant degradation in the first year of implantation, with an overall device duration of 2 to 3 years. The placement of the splint occurs after isolating the anterior and lateral portions of the trachea and/or main bronchi, where the malacic segments have been confirmed: Figure 19.

The two patients with bronchial splints were simultaneously subjected to cardiac surgery with the French maneuver (moving the pulmonary artery confluence anterior to the ascending aorta and reconstructing the right pulmonary artery), with evidence of good long-term results both clinically and in subsequent radiological and endoscopic controls.

The patient who underwent tracheal splint placement also showed a significant increase in respiratory space in radiological and endoscopic control with respiratory stabilization. However, six months after the procedure, the general condition of the patient with dystonic–dyskinetic tetraparesis, epileptic encephalopathy, and progressive pontine impairment necessitated the restoration of long-term ventilatory support with the creation of a tracheostomy.

In conclusion, the main advantage of treating TBM with bioresorbable splints is the reduction in endoluminal complications typical of endotracheal and endobronchial stents (dislocation, granulation, reduced mucosal clearance, and obstruction), allowing good radial expansion and resistance to external compression. Splint placement is generally performed during other cardiovascular surgery interventions, given the need for a sternotomy approach. Polycaprolactone and hydroxyapatite devices have a long duration, ensuring long-term stabilization with good adaptation to airway growth. The preliminary results reported in the literature and in our center are encouraging, but the indication is currently for “compassionate use,” still reserved for severe TBM cases.

### 7.2. The Tracheal Stent

Once the TM has been classified as primary/secondary and its severity, the most appropriate therapy can be proposed to the parents, choosing from the possible current therapeutic options [1]:

- AA, in case of extrinsic vascular compression;
- PT, in case of hypermobility of the pars mebranacea;
- Positioning of external tracheal splint;
- Positioning of endoluminal stents, which we will talk about.

The placement of a stent takes place in the operating/endoscopy room under double vision: endoscopic and radiological (the stents are radiopaque, either by their structure or thanks to the presence of metal markers), to achieve the best possible precision in the tracheal positioning of the device, although fine position adjustments are made with optical forceps [77,78].

The main complications related to the placement of a stent in the trachea are as follows:

- Dislocation: to prevent it, the stent diameter must be about 2 mm larger than the trachea’s diameter.
- Growth of granulation tissue and/or scar tissue at the edges of the stent, which occurs due to (1) stagnation of secretions triggering chronic inflammatory phenomena and (2) chronic ischemic damage related to the larger diameter of the stent at its edges (about 1–2 mm compared to the stent central section).
- Stagnation of catarrhal secretions inside the stent: this occurs mainly with silicone- and nitinol-coated stents because the stent, covering the tracheal mucosa along its entire length, cancels ciliary activity/motility, causing catarrhal stagnation inside it. With resorbable PDS stents, this problem occurs less frequently as they are open-mesh devices that do not completely abolish ciliary activity. It is important to promote expectoration, keep the airways humid, and keep the secretions well hydrated.

The golden rule of tracheal stenting is that, if TM is secondary to extrinsic vascular compression, the first treatment is the removal of the vascular compression. If a stent were placed in these patients, there would be a risk of causing ischemia of the tracheal wall, compressed by the vessel (especially if arterial) on the outside, and by the stent on the inside, with the risk of ischemic necrosis, fistulization, massive hemoptysis, and potentially fatal outcomes. After placing a stent inside the airways, it is essential to perform serial endoscopic follow-up with precise timing to prevent or anticipate the main complications described.

Until about 10–15 years ago, the following stents were mainly used [79,80]:

- Nitinol Stent: Self-expanding, made of a thermoplastic metal alloy coated in silicone that is not covered by the tracheal mucosa, so the stent is not incorporated into the tracheal wall. It needs to be periodically replaced, according to the child’s growth curve.
- Dumon Silicone Stent: Also self-expanding, but with a lower radial pressure than the nitinol stent. Currently, it is possible to produce a custom-made stent using a 3D printer, modeled after the measurement of the patient’s airways by CT scan.
- Stainless Steel Stent (BEMS): Usually pre-mounted on a balloon that needs to be inflated to dilate the device to its nominal size. This stent is not indicated for the treatment of TM because, while it is covered by the mucosa at the bronchial level and is no longer visible endoscopically after 4–6 months, it cannot be incorporated into the tracheal wall, and the high pressures to which the trachea is subjected can cause ovalization and, in the worst case, stent fracture/breakage.

An important turning point in the therapy of TM with the use of stents occurred about 15 years ago, when

- PDS (Polydioxanone) Stent entered the market. Being resorbable, they revolutionized tracheal stenting, replacing other types of stents [81,82]. Like the steel one, the PDS stent is a self-expanding open-mesh device, positioned with its own kit. It exerts radial support pressure for about 6–8 weeks, after which the resorption phase begins, lasting about another 8 weeks, occurring partly by lysis and partly by fragmentation. Therefore, it does not need to be removed, which is a great advantage as it avoids a potentially traumatic maneuver for the trachea, and the follow-up has been simplified and shortened in time. Another advantage is that, during the period of in situ permanence for about 8 + 8 weeks, it seems to help consolidate the tracheal wall with which it is in contact. PDS stents have a cost of up to about 3000 Euros, and this must be taken into account when planning conservative treatment. In conclusion, tracheal stenting, performed in centers specialized in advanced airway management, is a valid conservative treatment in the management of TM.

### 7.3. Indication for Decannulation in Tracheostomized Patients

The indication for tracheotomy in patients with TM is reserved for cases characterized by severe symptoms: cyanosis, severe obstructive apnea, recurrent infections, respiratory failure unresponsive to ventilation, and inability to extubate. The most recent literature shows that patients with TM treated with tracheotomy represent a small percentage of the larger case series, particularly regarding those who have undergone AA or PT [83,84,85]. For all of these patients, once the surgical therapeutic process is completed and the obstruction at the lower airways level is resolved, it is necessary to address the decannulation process, an iterative process that includes progressive steps until the removal of the tracheal cannula and the possible surgical closure of the stoma. This procedure involves the progressive reduction in the tracheal cannula’s diameter and temporary closure during the day under the caregiver’s close supervision. Subsequently, the cannula is closed even at night and monitored by oximetry/polysomnography, preferably during hospitalization in an intensive care setting. Finally, the tracheostoma is closed by surgical plastic surgery, followed by further monitoring in an intensive care setting [86].

The fundamental phases of decannulation are (1) the progressive down-sizing of the tracheal cannula and (2) its gradual closure, using a filter, then a phonatory valve, and finally a cap.

To ensure these phases are successful, it is necessary to verify the following aspects before starting decannulation:

- Identify the presence of other obstructive sites to take any further therapeutic measures.
- Evaluate the motility of the vocal cords (VC) and larynx.
- Assess the obstructive tracheal pathology and its resolution.

These key points can be evaluated with flexible transnasal endoscopy in an awake patient or, more commonly, in pediatric age, with laryngotracheoscopy under general anesthesia in spontaneous breathing.

Regarding possible endoluminal obstruction sites, a frequent cause of decannulation failure is peristomal endoluminal granulations, which, depending on their position and endoluminal extension, can be surgically treated using different techniques before decannulation [87].

The motility of the VC must always be evaluated, especially after surgical interventions involving the mediastinum, a surgical field where the recurrent nerves run, with the consequent possibility of VC paresis or paralysis. In the case of unilateral motility deficit, the first therapeutic decision consists of clinical and endoscopic follow-up associated with speech therapy rehabilitation. In the case of bilateral VC paralysis, endoscopic cordal laterofixation can be performed to increase the respiratory glottic space [88]. The removal of the tracheostomy is then deferred once the surgical dilation process of the respiratory glottic space is completed.

In conclusion, the fundamental steps of the decannulation procedure are

- The progressive reduction in the tracheal cannula’s diameter and temporary closure during the day under the caregiver’s close supervision;
- Respiratory monitoring by oximetry and polysomnography with the cannula closed even during sleep, which strongly indicates the possible removal of the tracheal cannula;
- Endoscopic re-evaluation of the airway, looking for any residual obstructive sites;
- The final multidisciplinary decision to decannulate the patient.

### 7.4. Indications for Prescribing N-Invasive Ventilation (NIV) in Patients with TM

The indications for NIV support in patients with TM/TBM, according to ERS recommendations [1], are limited to patients with recurrent infections and certain clinical conditions, particularly in (a) patients awaiting surgical intervention for TM, which in most cases consists of AA or PT; (b) patients in whom the surgical intervention was not resolutive, with persistent recurrent lower respiratory tract infections, presence of respiratory distress with obstructive events (e.g., ALTE-like episodes, retractions, etc.); (c) patients who have contraindications to surgical intervention due to severe comorbidities with high anesthetic risk, or in whom there are technical limitations (fortunately very rare) to the intervention itself; and (d) patients in whom it has been decided, in agreement with the family, to wait for the evolution of the clinical picture, before making a definitive therapeutic decision.

Continuous Positive Airway Pressure (CPAP) with a nasal interface is definitely indicated as a first approach, because it is preferable in terms of safety and comfort for the patient [89]: “Safety”, because the nasal interface reduces the risk of aspiration (in case of vomiting, for example); “comfort”, because, with a nasal interface, theoretically, the patient’s verbalization or the ability to take small amounts of liquids or food is not impeded; it is also less “oppressive” compared to a full-face interface. It should also be noted that, for patients under 2–4 years of age, the availability of different types of masks on the market is more limited compared to other age groups.

In most cases, low pressure values (even below 8–10 cm H_2_O) are sufficient to optimize ventilation, keeping the airways open. When higher pressures are needed, both to make ventilation more acceptable and to reduce the risk of gastric hyperinflation, a “bilevel” mode can be chosen [90].

Although there are no definitive data in the literature [91], the same concept of “pressure support” can be applied to respiratory physiotherapy with Positive Expiratory Pressure (PEP) techniques such as the PEP mask, which are used to improve secretion management and reduce the incidence of respiratory infections. Most studies in the literature refer to patients with cystic fibrosis, but in our clinical experience, both the PEP mask respiratory physiotherapy and the NIV prescription lead to a lower recurrence of infectious episodes in treated patients. This data obviously needs to be confirmed by “ad hoc” studies and can represent a stimulating starting point for the design of multicenter scientific works on the subject.

**Discussion**. The essential and definitive role of surgical treatment of TM was recognized by all participants, but the importance of other support techniques was also emphasized. This is clearly essential in patients who cannot undergo surgery, but also in those in whom respiratory assistance is intended as a “bridge solution” to accompany the patient to surgery. In patients with tracheostomy who undergo TBM surgery, the stoma can often be closed during surgery. When this does not occur, the procedure leading to subsequent removal of the tracheostomy tube must be standardized in its steps, according to a process shared by pediatric pulmonary centers. It was also recognized that the advent of NIV has made the indications for tracheostomy less frequent.

## 8. Conclusions

We have attempted to describe in this work, in the most complete and organic way, all the diagnostic and therapeutic aspects that must be addressed in the patient with TM. To summarize the meaning of our work, the take-home messages are as follows:

- Each patient must be evaluated individually, in their own specificity.
- The patient must be taken care of by a multidisciplinary team: a Tracheal Team, which must include pulmonologists, thoracic surgeons, cardiologists, otolaryngologists, radiologists, gastroenterologists, and anesthesiologists for case-by-case therapeutic decisions, all experts in the care of TM patients.
- The clinical history and CT scan with CM contribute to the overall evaluation of the individual patient, and the role of DVBS is essential: the endoscopy must also include a phase in which the patient twists their abdomen and tries to cough, with a consequent increase in intrathoracic pressure. It can thus be verified whether the main pathology, causing tracheal malacia, affects the anterior or posterior tracheal wall; it is therefore possible to decide whether the patient will benefit from the anterior aortopexy operation, in case of extrinsic compression from an arterial vessel, or posterior tracheopexy, if there is an abnormal hypermobility of the pars membranacea.
- When TM is present and severe, but the patient cannot undergo surgical treatment, it is also essential that the patient can benefit from all other respiratory assistance aids: insertion of tracheal splint and stent, placement of tracheostomy, and, hopefully, once the surgical therapeutic process is completed and the obstruction at the lower airways level is resolved, address the decannulation process.
- We must also consider how, in recent decades, the increasingly widespread use of non-invasive ventilation (NIV) has made it increasingly less necessary to resort to tracheostomy.

In conclusion, our hope is that this meeting and this resulting article, which is the summary of a multicentric experience in Italy, as stated in the title, may lead to increasingly shared diagnostic and therapeutic pathways, up to the formulation of guidelines recognized by the various pediatric centers that care for patients with TM, both in Italy and abroad.

## Data Availability

The original contributions presented in this study are included in the article. Further inquiries can be directed to the corresponding author.

## Abbreviations

AA: Anterior Aortopexy, ALTE: Apparent Life-Threatening Event, APD: Anteroposterior Diameter, BAL: Bronchoalveolar Lavage, CM: Contrast Medium, CPAP: Continuous Positive Airway Pressure, CT: Computed Tomography, CXR: Chest Radiography, DCT: Dynamic CT, DVBS: Dynamic Videobronchoscopy, EA: Esophageal Atresia, EG-EF: Esophagography–Fluoroscopy, FEV1: Forced Expiratory Volume in 1 Second, F/VC: Flow/Volume Curve, HFNC: High Flow Nasal Cannula, IA: Innominate Artery, MIS: Mini Invasive Surgery, MRI: Magnetic Resonance Imaging, NIV: Non Invasive Ventilation, OAA: Open Anterior Aortopexy, PBB: Protracted Bacterial Bronchitis, PEF: Peak Expiratory Flow, PEP: Positive Expiratory Pressure, PFT: Pulmonary Function Test, PT: Posterior Tracheopexy, RATS: Robotic-Assisted Thoracoscopic Surgery, TAA: Thoracoscopic Anterior Aortopexy, TBG-TBF: Tacheobronchography–Fluoroscopy, TBM: Tracheobronchomalacia, TEF: Tracheoesophageal Fistula, TM: Tracheomalacia, TMCS: Tracheomalacia Clinical Score, TMES: Tracheomalacia Endoscopic Score, TMS: Tracheomalacia Score, TT: Tracheal Team, SVBS: Static Videobronchoscopy, VATS: Video-Assisted Thoracoscopic Surgery, VBS: Videobronchoscopy, VCs: Vocal Cords.
